# NemoProfile as an efficient approach to network motif analysis with instance collection

**DOI:** 10.1186/s12859-017-1822-6

**Published:** 2017-10-16

**Authors:** Wooyoung Kim, Lynnette Haukap

**Affiliations:** Division of Computing and Software Systems, School of Science, Technology, Engineering, and Mathematics (STEM), University of Washington Bothell, 18115 Campus Way NE, Bothell, 98011-8246 WA USA

**Keywords:** NemoProfile, NemoCollect, ESU, Systems biology, Biological network, Network motif, Essential protein

## Abstract

**Background:**

A network motif is defined as a statistically significant and recurring subgraph pattern within a network. Most existing instance collection methods are not feasible due to high memory usage issues and provision of limited network motif information. They require a two-step process that requires network motif identification prior to instance collection. Due to the impracticality in obtaining motif instances, the significance of their contribution to problem solving is debated within the field of biology.

**Results:**

This paper presents NemoProfile, an efficient new network motif data model. NemoProfile simplifies instance collection by resolving memory overhead issues and is seamlessly generated, thus eliminating the need for costly two-step processing. Additionally, a case study was conducted to demonstrate the application of network motifs to existing problems in the field of biology.

**Conclusion:**

NemoProfile comprises network motifs and their instances, thereby facilitating network motifs usage in real biological problems.

## Background

**Fig. 1:**
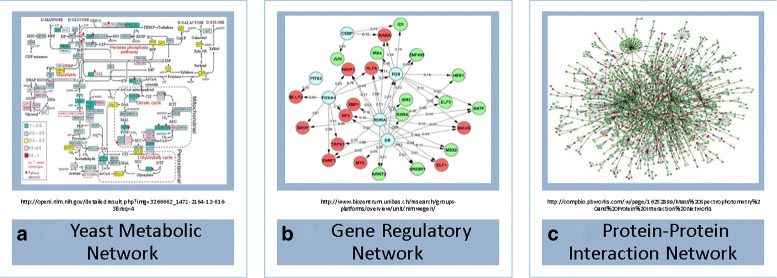
Examples of biological networks: **a** a metabolic network is composed of different types of nodes and edges; **b** all the nodes in a gene regulatory network are genes, and directed edges represent a regulatory process; **c** a protein-protein interaction network is composed of proteins, and their binary interactions are undirected edges

Systems biology elucidates, models, and predicts the behavior of all biological components and their interactions. Its emphasis on the interconnections of molecules produced biological networks as described in Fig. 1, where nodes are molecules and edges are interactions between them. Understandably, various graph theory topics are substantially applied to resolve various biological problems, such as prediction of biological function, detection of protein complexes, discovery of new interactions, evolutionary analysis, information integration, diagnosis of disease, and drug design [1].

Network motif analysis is one of the graph theory methods used to find biologically relevant functions in networks [2]. A network motif is defined as an overly frequent and unique subgraph pattern in a network, and it has been applied to solve various biological and medical problems: predicting protein-protein interactions [3], determining protein functions [4], detecting breast-cancer susceptibility genes [5], investigating for evolutionary conservation [6, 7], and discovering essential proteins [8, 9]. Furthermore, a broad spectrum of applications has been explored: ‘motif clustering’ [10], ‘motif themes’ [11], ‘relative graphlet frequency distances’ [12, 13], ‘motif modes’ [14], and ‘MotifScores’ [15].

However, identifying network motifs is intrinsically very costly, and this high computational cost restricts extensive and exhaustive experiments for real problems. The process involves enumeration of millions of subgraphs in the input graph, and classification through canonical labeling or isomorphic testing. Then, a network motif’s uniqueness is established through rigorous statistical testing in a huge random pool. Consequently, various heuristic methods and parallel algorithms have been proposed that alleviate the performance concerns of exhaustive search methods [16].

Network motifs may remain meaningless unless their biological significance is properly evaluated. In order to determine biological relevance, individual motif instances need to be collected and evaluated in the context of biological systems. However, most motif-finding algorithms provide only frequency and statistical significance of each pattern, which restricts its usability for real-world problems. Therefore, we introduce a new network motif representation to overcome this problem, and define it as ***NemoProfile***.

In this paper, we show how efficiently NemoProfile is generated and how this significantly reduces motif instance collection time. We also provide a case study where NemoProfile is directly applied to the prediction of essential proteins from protein-protein interaction (PPI) networks.

## Methods

**Fig. 2:**
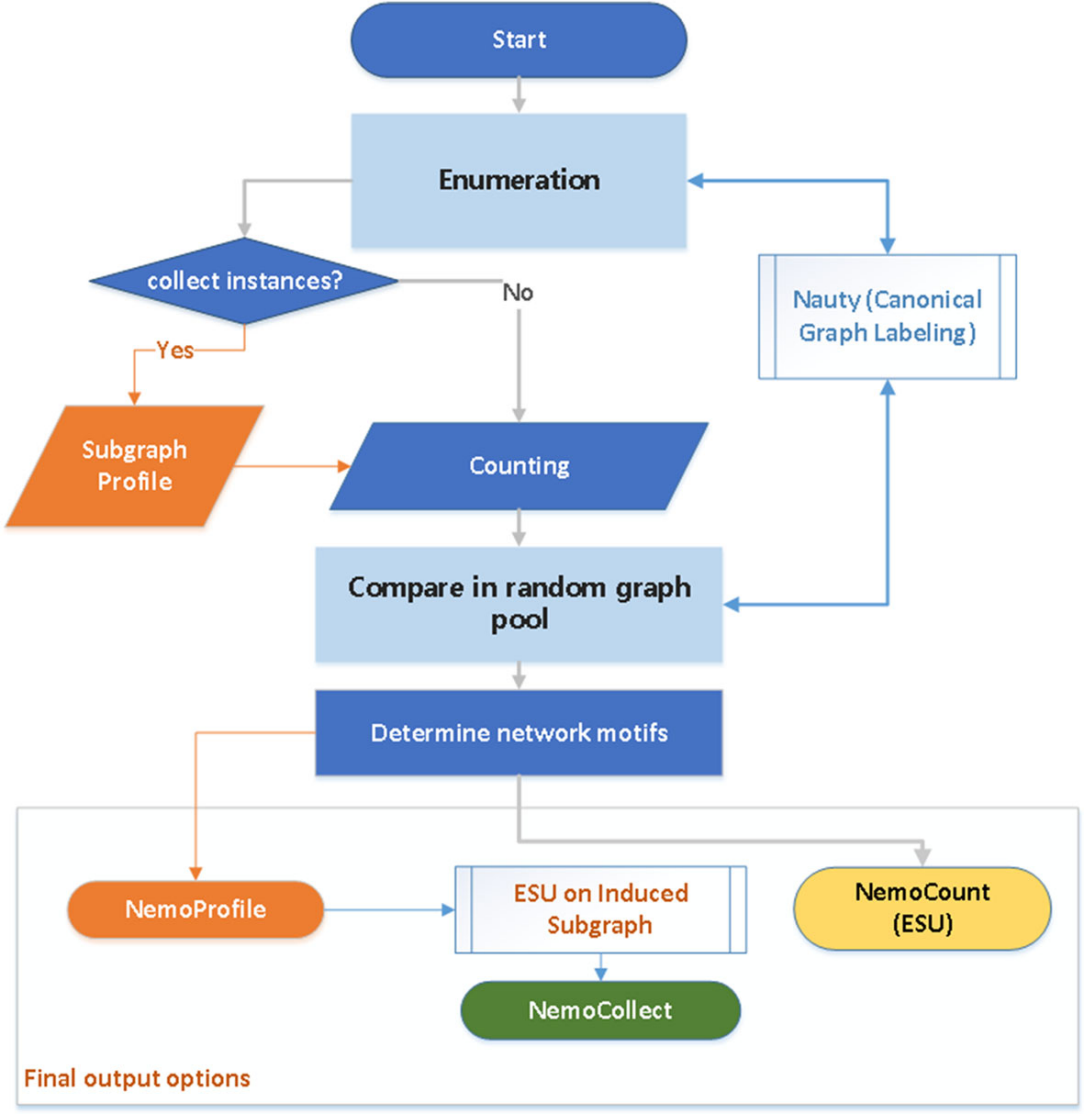
Flow chart of a network motif finding program producing NemoProfile, NemoCollect, and NemoCount(ESU). It searches all subgraphs in a given network by ‘Enumeration,’ then asks if instances should be collected. If yes, all the instances are collected as ‘SubgraphProfile’ form. Otherwise the occurrences of each graph pattern is recorded as in the ‘Counting’ step. These counts are essential to determine which graph pattern is a network motif as its relative frequency is compared in the random graph pool. In the final output, the SubgraphProfile is used to produce NemoProfile or NemoCollect. NemoCount output can be produced without the SubgraphProfile

Here, we introduce a new network motif representation, as ***NemoProfile***. NemoProfile can be effortlessly generated while detecting network motifs, and effectively collects network motif instances. We designed and implemented a program based on a flowchart illustrated in Fig. 2 to provide three separate output options: NemoProfile, NemoCount, and NemoCollect.

NemoCount, which implements ESU (Enumerate SUbgraphs) algorithm [17], provides the frequency and statistical testing result only. NemoProfile and NemoCollect are described followed by the definition of network motifs.

### Network motif

Network motifs are defined as frequent and unique subgraphs in a network. Formally, if *G*=(*V*,*E*) is a graph and *k* ranges from 3 to *n*<<|*V*|, then a **network motif** *m* is a connected subgraph of size *k* in *G*, which appears more frequently than usual. In the definition of network motifs, ‘more frequent than usual’ refers to a structural uniqueness and it is determined by *p*-value as in Eq. (1) or *z*-score as in Eq. (2) after a number of random graphs have been generated.

1 $${} {{p \text{-value}(m) = \frac{1}{N} \sum\limits_{n=1}^{N} c(n), \text{where~} c(n)= \left\{ \begin{array}{ll} 1, & \text{if}\; f_{R}(m) \geq f_{G}(m) \\ 0, & \text{otherwise}.\\ \end{array} \right.}}  $$

2 $$ z \text{-score}(m) = \frac{f_{G}(m) - average (f_{R}(m))}{std(f_{R}(m))}  $$

Here, *f*_*G*_(*m*) is the frequency of motif *m* in *G* and *f*_*R*_(*m*) be that of motif in random graph *R*. Also, *a* *v* *e* *r* *a* *g* *e*(*f*_*R*_(*m*)) and *s* *t* *d*(*f*_*R*_(*m*)) refer to the average and standard deviation of frequencies in random networks, respectively. Generally, a subgraph with *p*-value less than 0.01 or *z*-score greater than 2.0 is considered as a network motif.

**Fig. 3:**
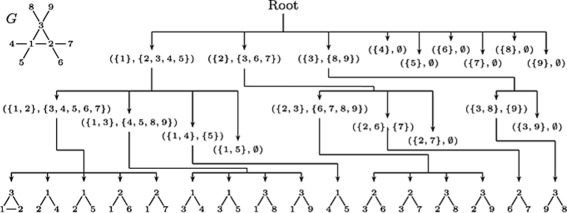
An example of a graph with network motifs and their instances, courtesy of paper [17]

Figure 3 describes how to find size 3 network motifs from the input graph *G* in the upper left corner by ESU algorithm [17]. The method enumerated a total of 16 subgraphs of size 3, but one instance, ({1,2,3}), is a triangle type while others are all linear types. Although the frequency of triangle type is much less than the linear type, *p*-value and z-score determine that the triangle type is a network motif. Therefore the frequency (or count) of the network motif is 1, and the instance of the network motif is ({1,2,3}). We want to note that all existing software programs provide the frequency, and *p*-value or z-scores of network motifs but not the instances of network motifs due to heavy memory overhead. In this paper, we put more weight on the importance of network motif instances by introducing a NemoProfile.

### Network motif detection algorithms

**Fig. 4:**
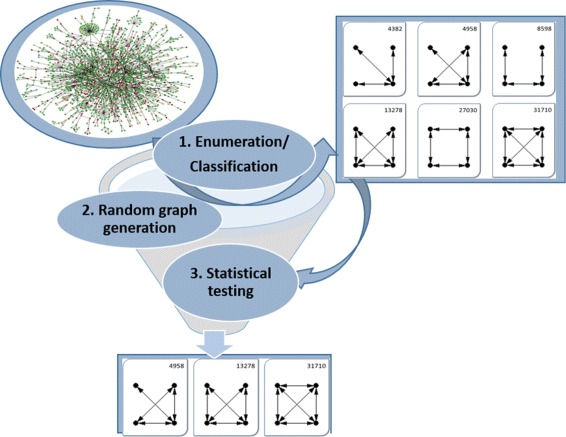
Network-centric methods consists of enumeration and classification, random graph generation, and statistical testing. For example, out of six non-isomorphic subgraphs of size 4 (upper-right table), three patterns at the bottom are determined as network motifs

Various network-motif-finding algorithms are available, classified into network-centric and motif-centric algorithms [16]. Network-centric algorithms identify network motifs while exploring subgraphs in the input graph, whereas motif-centric algorithms count the instances for each pattern in a predefined query set. Then its significance is determined through various statistical testing in a large random pool to determine network motifs, as summarized in Fig. 4.

Although network-centric algorithms have the benefit that subgraphs that are not in the input graph will never be considered, the inevitable enumeration process is heavily expensive. Motif-centric algorithms can reduce classification time if combined with symmetry breaking or mapping strategies, and can directly verify whether a specific pattern is a network motif or not [16, 17]. However, the number of non-isomorphic subgraphs (patterns) increases exponentially with the size of motifs, therefore even listing them is intractable. As an example, there are 11,716,571 patterns for motif size 10, as shown in Table 1

**Table 1:** Number of non-isomorphic subgraphs for undirected and directed graphs with up to 10 vertices [31]

Vertices	Number of non-isomorphic subgraphs	
	Undirected	Directed
1	1	1
2	1	2
3	2	13
4	6	199
5	21	9,364
6	112	1,530,843
7	853	880,471,142
8	11,117	1,792,473,955,306
9	261,080	13,026,161,682,466,200
10	11,716,571	341,247,400,399,400,000,000

**Fig. 5:**
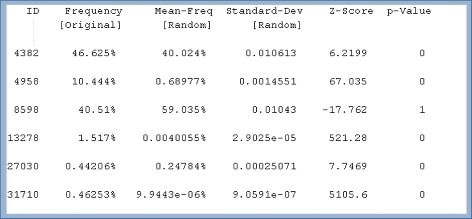
The example format of network motif finding outputs, which shows frequency and significance for each pattern

Many motif search programs are also available [18]: MFinder [19], FANMOD [20], Kavosh [21], Mavisto [22], and NeMoFinder [23] follow the network-centric methods. Motif-centric methods are available with Grochow’s [24], and MODA [25]. However, most of them provide only frequency and statistical significance as in Fig. 5, because collecting all instances of each pattern creates a serious memory overhead problem. Hypothetically, the number of subgraphs in the input graph is $|E_{G}|^{|E_{m}|}\phantom {\dot {i}\!}$ where |*E*_*G*_| is the number of edges in the input graph and |*E*_*m*_| is the number of edges in motif *m* [26]. That means most biological networks have several tens or even hundreds of millions of subgraphs, even for small motifs. Therefore, instances of network motifs have to be collected as post-processing if necessary, and it usually requires more efforts than detecting network motifs, as this step is unavailable with current programs.

Considering that most real world problems that use network motifs require a knowledge of what nodes and edges actually belong to network motifs [8, 9], providing their instances will greatly increase the usability of network motifs. Therefore, the work in this paper focuses on the neglected task in network motif finding, which is collecting instances efficiently and utilizing them for real biological problems.

### NemoProfile

To reduce computational cost but still provide valuable results, we propose a new network motif representation, **NemoProfile** that relates each node to network motifs as a profile matrix while identifying network motifs. As illustrated in Fig. 2, a *SubgraphProfile*, ***T*** as an *n*×*m* matrix is first constructed where *n* is the number of nodes in the input graph and *m* is the number of all subgraph patterns of size *k*. While enumerating, *T*_*ij*_ increments by 1 if a pattern *m*_*j*_ includes node *v*_*i*_. After network motifs are determined NemoProfile takes the network motif columns from *T*.

**Fig. 6:**
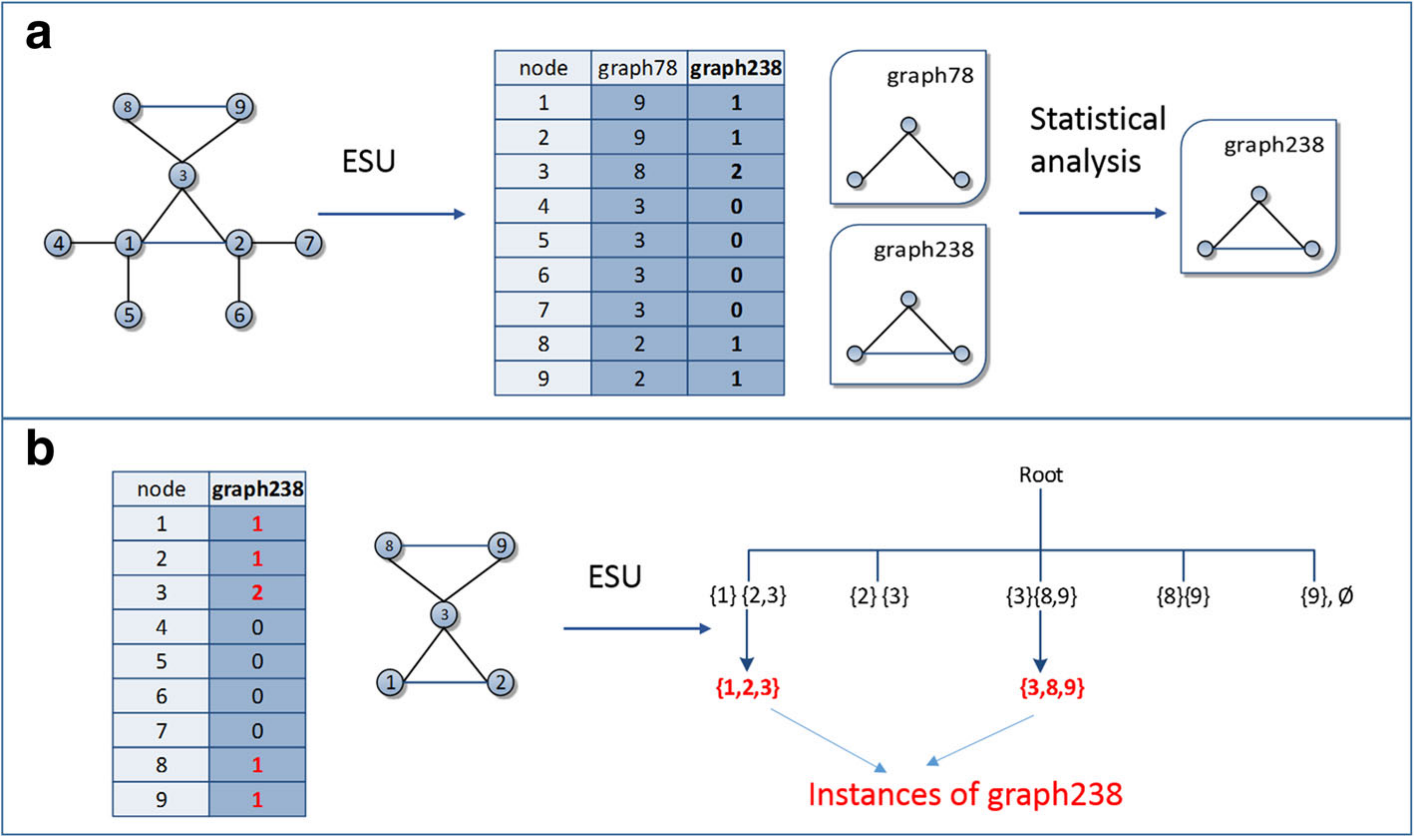
The process of collecting instances for graph238: **a** SubgraphProfile is obtained in the process of ESU from the original input graph in the left. From the graph patterns graph78 and graph238, “graph238” is determined to be a network motif through statistical analysis; **b** NemoProfile (left) is derived from the SubgraphProfile in (**a**), and effectively identifies an induced subgraph for motif “graph238” by collecting the nodes corresponding to a non-zero value in the column of graph238. The induced subgraph with nodes 1, 2, 3, 8, and 9 will be processed with *EnumerateSubgraph*(ESU) [17] to collect all the instances for graph238

For example, we can find 14 instances of “graph78” size-3-subgraphs and 2 of “graph238” size-3-subgraphs if a target graph has 9 vertices and 10 edges as shown in Fig. 6a. While the Fanmod program that implements ESU can trace all 16 instances, saving all instances as sets of vertices, such as, 〈 graph78 = ({1, 2, 4},{1, 2, 5}, {1, 2, 6},...,{2, 3, 9}), graph238 =({1, 2, 3}, {3, 8, 9}) 〉 causes a great amount memory overhead. Therefore, Fanmod (ESU algorithm) provides the frequencies and statistical results of each type, but discards the motif instances. On the other hand, NemoProfile saves the set of instances as a matrix so that the frequency of each node’s involvement to each pattern is recorded as shown in Fig. 6a. Figure 6b describes how to recover the network motif instances as sets of vertices from a NemoProfile, which generates NemoCollect.

### NemoCollect

We define *NemoCollect* as in Algorithm 1 describing the process to collect instances of a network motif *m* with NemoProfile. It derives an induced subnetwork comprised by all nodes whose *m*-corresponding column value being non-negative. The subnetwork is fed back to *EnumerateSubgraph* function from [17] to collect the instances of motif *m*. Figure 6 illustrates the process with an input graph and NemoProfile.



## Results and discussion

We tested the efficiency and effectiveness of NemoProfile with a number of PPI networks that are available in the DIP database [27] in a Linux operating system, Xeon Core i7 with 5,959 MiB system memory. The DIP database includes eight different species of protein-protein interaction (PPI) networks, which are manually and computationally curated. Almost every three months, the networks are updated by adding or removing proteins and their interactions. We selected five E. coli core PPIs, five S. cerevisiae core PPIs, and six H. sapiens core PPI networks that are updated each year from 2010 through 2014.

We designed and implemented a program by modifying ESU with SNAP (Stanford Network Analysis Platform) C++ library [28] to have three separate output options, ‘NemoCount (ESU)’, ‘NemoProfile’, and ‘NemoCollect’ as shown in the bottom box in the flowchart of Fig. 2. We should note that the ‘SubgraphProfile’ is an intermediate datum to generate ‘NemoProfile’ and ‘NemoCollect’ at the end.

**Fig. 7:**
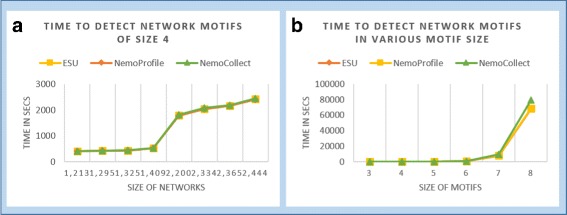
**a** The chart shows that the running time (Y-axis) to detect size 4 network motifs from various size (number of nodes, X-axis) of PPI networks is very similar with all three options; **b** This chart compares times of ESU, NemoProfile, and NemoCollect methods while detecting various sizes of motifs, from size 3 to size 8. NemoCollect takes longer than the other options for larger motif sizes

Performances of NemoProfile and NemoCollect are compared with NemoCount in various testing scenarios by varying the size of the input graph, or by varying the size of network motifs to detect. Figure 7a demonstrates *NemoProfile* and *NemoCollect* take almost the same time as *NemoCount*(ESU) for size 4 network motifs in various input graph sizes. Time for detection of various sizes of network motifs is also compared in Fig. 7b. Inevitably, NemoCollect takes slightly more time than others as the size increases due to the additional instance collection time. However, time of NemoProfile is still similar to that of ESU proving that it is efficiently generated but contains much richer information than ESU.

**Fig. 8:**
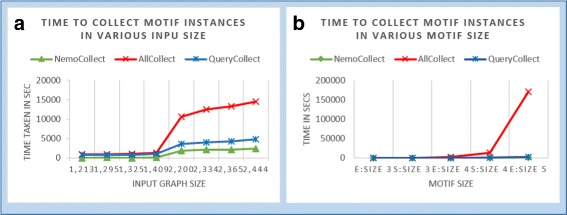
**a** This chart shows that NemoCollect takes significantly less time compared to AllCollect and QueryCollect, while collecting size 4 motif instances from various inputs; **b** It shows that AllCollect takes up significant time compared to other methods, and that NemoCollect method is the most efficient when collecting size 5 motif instances

Next, we wanted to see if NemoProfile significantly alleviates the memory overhead problem when collecting network motif instances. We design “NemoCollect” process as shown in Algorithm 1 which uses NemoProfile in the process. Since none of the existing network motif finding algorithms collect network motif instances, we designed a couple of alternatives to compare them with NemoCollect: **AllCollect** is collecting all subgraphs while searching network motifs, and **QueryCollect** is collecting the instances of motifs using motif-centric method. Although the time of AllCollect is directly measured, the time for QueryCollect method is estimated, assuming that it will run ESU first to determine network motifs and run MODA later to collect the instances of the network motifs. Since MODA takes as much time as ESU, according to paper [25, 26], we estimated the time for QueryCollect as twice that of ESU. Figure 8a and b demonstrate that NemoCollect is the most efficient method for motif instance collection, even with an increase in motif size. Table 2 supplements Fig. 8b to show the differences clearly.

**Table 2:** Running time (in seconds) of NemoCollect, QueryCollect and AllCollect with various motif sizes, as in Fig. 8b

	*E:size3*	*S:size3*	*E:size4*	*S:size4*	*E:size5*
NemoCollect	5.56	27.86	61.74	348.01	1768.40
QueryCollect	11.18	54.14	117.45	655.04	2504.40
AllCollect	17.74	159.53	2405.90	131.49	170,420.00

NemoCollect is the most efficient, while AllCollect becomes intractable with motifs of larger sizes

### Case study: essential protein prediction and NemoProfile

This section demonstrates the usability of NemoProfile for real-world applications, specifically predicting essential proteins in a PPI network where network motif analysis has been applied previously [8, 9]. We used E. coli (‘Ecoli20101010CR’) and S. cerevisiae (‘Scere20101010CR’) PPI networks from DIP, and obtained the list of essential proteins from *Database of essential genes (DEG)* [29]. E. coli has 121 essential proteins out of 1,231 nodes, and S. cerevisiae contains 782 essential proteins out of 2,200 proteins.

First, NemoProfile program provides the NemoProfile matrix (*A*) of each network where the number in *A*_*ij*_ refers the number of protein *i* overlaps with a motif *j*. Here, five network motifs are identified in both of the networks, and NemoProfile structure is directly converted to the set of attributes for each protein. The data attributes along with the protein’s essentiality is fed into Weka program [30] to run a decision tree (J48) algorithm to predict essential proteins.

**Fig. 9:**
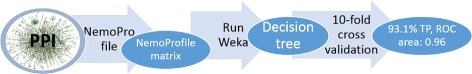
Process to predict essential proteins in a PPI network. A PPI network is processed to obtain NemoProfile matrix (*A*) that shows protein *i* has *A*_*ij*_ overlaps with motif *j*. The matrix is directly loaded into a Weka program to run a decision tree algorithm and is evaluated through 10-fold cross-validation method to provide prediction rate and ROC area value as a result

**Fig. 10:**
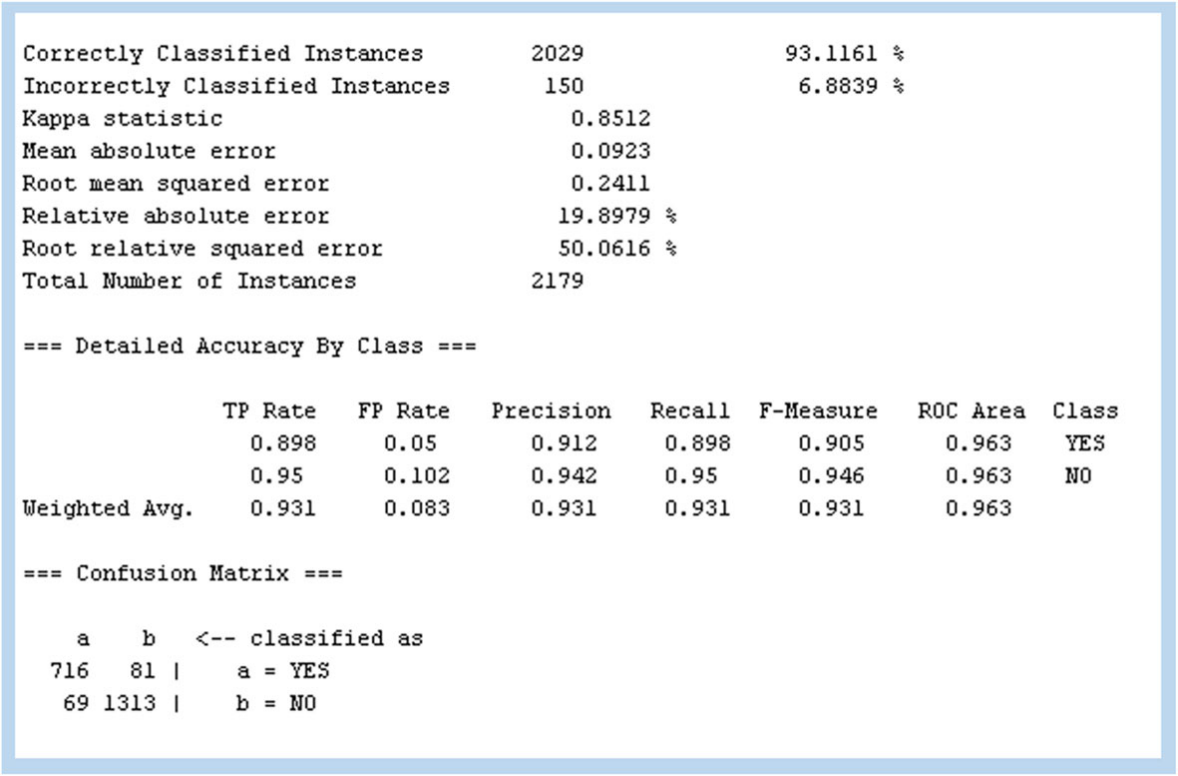
Result of prediction of essential proteins with Weka, detected from S. cerevisiae

Figure 9 summarizes the overall process, from a PPI network, through NemoProfile, and the application of the decision tree technique to predict essential proteins of an organism. The classification is evaluated using 10-fold cross-validation scheme, and Fig. 10 is one example of Weka results on S. cerevisiae PPI.

## Conclusions

Several computationally costly tasks are required for network motif finding since network motifs are unique both structurally and statistically. These tasks include enumeration, classification, and statistical analysis. Network-centric and motif-centric methods exist for finding motifs. While these methods have reduced computational costs, they have not overcome the prejudice towards network motifs in problem solving. The doubtfulness as to the relevance of network motifs in biological problems continues due to the lack of usability with existing programs.

Therefore, we emphasized their usability by presenting NemoProfile, an efficient network motif representation. Significant improvement is seen with the memory overhead problem resolution and the reuse of NemoProfile to collect instances of motifs for direct application to existing problems. Additionally, NemoProfile provides the output from other representations, including the frequencies and statistical significance of subgraph patterns.

A NemoProfile program was constructed and used to demonstrate the effectiveness of network motifs in application to real world problems. The experiment was conducted using PPI networks and the results showed that NemoProfile succinctly represents network motifs and their instances with no extra computational costs incurred. With a favorable outcome in comparison with other alternative methods NemoCollect is defined as the process of collecting instances from NemoProfile. The outcome clearly demonstrates that the performance is significantly better than the alternatives. A usability focused case-study of NemoProfile was performed to predict essential proteins in PPI networks. According to the study, the application of machine learning algorithms can be easily applied to NemoProfile by first converting it to data feature space.

Future works on NemoProfile include three main tasks. First, the design of a framework to enhance the application of NemoProfile to current and future problems, thus reducing prejudice towards network motif analysis in the field of biology. Second, enhance the NemoCollect process using parallelization by leveraging each separate column in NemoProfile. Third, improve the NemoCollect process using a symmetry breaking or mapping process.

## Abbreviations

DEG: Database of essential genes
DIP: Database of interacting proteins
ESU: Enumerate SUbgraphs
NemoCollect: Network motif instance collection
NemoCount: Network motif count
NemoProfile: Network motif profile
PPI: Protein-protein interaction
SNAP: Stanford network analysis platform
